# Triptolide alleviates psoriasis through inhibiting the Wnt5a/β-Catenin signaling pathway

**DOI:** 10.3389/fphar.2025.1534118

**Published:** 2025-04-29

**Authors:** Eryang Chen, Lei Wang, Qu Wang, Yan Cai, Yaning Dou, Hongyan Qu, Junyi Zhu, Haiyang Zhao, Suqing Zheng, Chengguang Zhao, Bin Chen

**Affiliations:** ^1^ The Affiliated Cangnan Hospital, Wenzhou Medical University, Wenzhou, Zhejiang, China; ^2^ School of Pharmaceutical Sciences, Wenzhou Medical University, Wenzhou, Zhejiang, China; ^3^ The Institute of Life Sciences, Wenzhou University, Wenzhou, Zhejiang, China

**Keywords:** psoriasis, triptolide, interleukin-17A, Wnt5a/β-catenin, inflammation

## Abstract

**Background:**

Psoriasis, an immune-mediated chronic inflammatory skin disease, is characterized by keratinocyte proliferation and inflammatory cell infiltration. *T* ripterygium wilfordii is a potential treatment option for psoriasis, and triptolide (TP) is one of its active components. TP may possess the potential to treat psoriasis; however, its mechanism of action remains unknown.

**Objective:**

The research aims to explore the therapeutic effect of TP on psoriasis and elucidate its potential targets.

**Methods:**

The imiquimod-induced psoriasis-like lesion mouse model was used to identify the mechanism underlying the therapeutic effect of TP.RNA-seq strategy was utilized to forecast the targets and mechanisms of TP in the context of psoriasis.Finally, we verify the effect of TP in the IL-17A-induced keratinocyte hyperproliferation and inflammation model.

**Results:**

TP reduced epidermal hyperplasia as well as psoriasis area and severity index scoring. Moreover, treatment with TP inhibited IMQ-induced splenomegaly and T-helper 17 cell differentiation in the psoriatic mice. Additionally, the treatment reduced the serum levels of pro-inflammatory cytokines such as interleukin (IL)-17A, IL-22, IL-23, IL-6, and tumor necrosis factor-α in the mice. The sequencing of RNA obtained from skin lesions of the psoriatic mice indicated that treatment with TP significantly downregulated Wnt5a RNA levels. Moreover, the Wnt5a/β-catenin pathway upregulated by IMQ was downregulated by treatment with TP. Additionally, IL-17A induced and upregulated Wnt5A and β-catenin mRNA expression, and TP inhibited this upregulated expression in HaCaT cells. Furthermore, TP inhibited proliferation, promoted apoptosis, and arrested the cell cycle in the IL-17A-induced keratinocyte hyperproliferation and inflammation model, thereby exhibiting its anti-inflammatory properties.

**Conclusion:**

TP alleviated psoriasis in mice by exerting anti-inflammatory effects and inhibited keratinocyte proliferation, which was partly achieved by regulating the Wnt5a/β-catenin signaling pathway.

## 1 Introduction

Psoriasis is characterized by excessive keratinocyte proliferation and immune cell infiltration and is manifested as erythema scale-like skin plaques (Ferrara et al., 2024; Yamanaka et al., 2021). However, the exact etiology and pathogenesis of the disease remain unclear. The interleukin (IL)-23/T-helper 14 cells (Th17) axis plays a central role in psoriasis pathogenesis. IL-17A is primarily secreted by Th17 cells, and one of its primary targets is keratinocytes, which are stimulated to over-proliferate, hyperkeratosis, and secrete chemokines (Di Cesare et al., 2009; Kim and Krueger, 2017; Mease, 2015). Furthermore, keratinocytes probably participate in eliciting and amplifying inflammatory responses, and this crosstalk between keratinocytes and immune cells results in psoriasis-like clinical lesions of the skin (Bugaut and Aractingi, 2021; Garzorz-Stark and Eyerich, 2019).

Many inhibitors developed against psoriatic cytokines have shown promising results; for example, secukinumab and ixekizumab targeting IL-17A and brodalumab targeting the IL-17A receptor have been approved by the Food and Drug Administration for treating moderate-to-severe psoriasis (Ferrara et al., 2024; Galluzzo et al., 2016; Hawkes et al., 2018). However, psoriasis is still considered incurable and such biologic-based therapies have some disadvantages such as they raise the risk of infection, are expensive, and require maintenance dosing; thus, many patients cannot afford them (Bian et al., 2021; Doherty et al., 2008; Pender and Kirby, 2024). Therefore, it is imperative to develop cost-effective and safer psoriasis treatment strategies based on the remarkable therapeutic effects of traditional Chinese medicine (TCM) (Huang et al., 2019).

Triptolide (TP), a diterpenoid, is a TCM monomer extracted from *Tripterygium wilfordii* (Chen et al., 2018) and exerts effective immunosuppressive and anti-inflammatory effects (Brinker et al., 2007). Additionally, TP has been proposed as a possible treatment option for psoriasis; however, the mechanism underlying its anti-psoriasis activity is unknown. Moreover, *in vivo* investigations conducted on patients or model mice with psoriasis are scarce. Thus, herein, we treated imiquimod (IMQ)-induced psoriatic mice with TP to evaluate its therapeutic effect and elucidate the underlying mechanism. We found that IMQ treatment-induced and upregulated Wnt5a expression in the model mice, and treatment with TP downregulated this expression.

Wnt5a, one of the activators of the Wnt signaling pathway, regulates cell proliferation, polarity, migration, differentiation, and inflammation (Zhang et al., 2024; Kim et al., 2010; Pashirzad et al., 2017). Reischl et al. (2007) found that Wnt5a was more expressed in the skin lesions of patients with psoriasis. By performing RNA sequencing and comprehensive analysis of skin samples, Dou et al. (2017) suggested Wnt5a as a key pathogenic gene associated with psoriasis and a probable biomarker for psoriasis diagnosis and treatment. The treatment of keratinocytes with recombinant human Wnt5a resulted in a pro-proliferation effect and increased the secretion of IL-23, IL-12, and tumor necrosis factor (TNF)-α *in vitro* (Kim et al., 2016)*. Conversely,* Wnt5a knockdown inhibited the proliferation and promoted the apoptosis of normal human keratinocytes and HaCaT cells. Additionally, the expression of β-catenin, an important gene downstream of Wnt5a, was suppressed (Zhang et al., 2015). In summary, activated Wnt5a signaling in psoriasis induces keratinocyte proliferation and cytokine secretion, which further regulate Wnt5a expression and pathway activation. This crosstalk leads to a signaling loop that promotes psoriasis development and progression (Tian et al., 2019). No studies have focused on the relationship between IL-17A and Wnt5a in psoriasis; however, Fabien Lavocat et al. showed that IL-17A stimulated Wnt5a expression in fibroblast-like synoviocytes (Lavocat et al., 2016). When we treated HaCaT cells with IL-17A, Wnt5a mRNA and protein levels increased. *In vivo* (animal) experiments performed to evaluate the anti-psoriasis effect of TP showed that TP regulated Wnt5a. Furthermore, we investigated the relationship between Wnt5a and IL-17A in psoriasis to provide a novel theoretical basis for the clinical application of TP and new targets for psoriasis treatment.

## 2 Materials and methods

### 2.1 Antibodies and reagents

Triptolide (#38748–32–2), methotrexate (MTX, #133073–73–1) was gained from Aladdin (Shanghai, China). Imiquimod was gained from Sichuan Mingxin Pharmaceutical Co. (Sichuan, China). Recombinant Human IL-17A Protein were ordered from PeproTech Co. (200–17–100, Rocky Hill, United States). Antibodies of PCNA (#10205-2-AP), CCD1 (#26939-1-AP), GAPDH (#10494-1-AP) were ordered from Proteintech co. (Wuhan, Hubei, China). Primary antibodies against Wnt5a (#ab227229), β-Catenin (#ab32572), Bax (#ab32503) and Caspase-3 (#ab32351) were ordered from Abcam Co. (Cambridge, MA, United States). Primary antibody against Bcl-2 (#sc-7382) was gained from Santa Cruz Biotechnology Inc. (Dallas, TX, United States). The secondary anti-rabbit IgG and anti-mouse IgG were gained from Santa Cruz Biotechnology Inc. (Dallas, TX, United States). Antibodies of FITC-conjugated CD4 (#11–0041–81), PE-conjugated IL-17A (#12–7177–81) were ordered from Invitrogen Life Technologies (Carlsbad, CA, United States). Elisa Kits (IL-17A, IL-22, IL-23, IL-6, and TNF-α) and SYBR Green and 2 × Taq PCR MasterMix were gained from Solarbio Science & Technology Co. (Beijing, China). Red Blood Cell Lysis Buffer was purchased from Beyotime Co. (Shanghai, China). Cell Stimulation Cocktail (#00–4970–93), TRIzolTM Reagent, hoechst 33,342 and Propidium Iodide (#V23201) were ordered from Invitrogen Life Technologies (Carlsbad, CA, United States). Propidium Iodide and FITC Annexin V Apoptosis Detection Kit I and Mouse sero block FcR (#553141) were ordered from BD Pharmingen (Franklin Lakes, NJ, United States).

### 2.2 Mice

The experiments were carried out with the approval of the Animal Policy and Welfare Committee of Wenzhou Medical College (No. XMSQ 2021–0021). Female BALB/c mice (6–8 weeks old) were purchased from Vital River Laboratory Animal Technology Co. (Beijing, China) and housed in the Animal Experimental Center of Wenzhou Medical University. Adaptatively reared mice were divided randomly into five groups of equal size, and the mice were anesthetized with CO_2_ and sacrificed at the end of the experiment.

### 2.3 Psoriasis-like model and triptolide treatment

All mice (7–9 weeks) were randomly assigned to control group (n = 7), IMQ group (n = 7), positive control group (n = 7), low dose triptolide group (n = 7), and high dose triptolide group (n = 7). According to a previous report (Van der fits et al., 2009), mice whose backs were shaved and rested for a day, Then 62.5 mg of 5% IMQ was applied daily to their shaved back, and control mice were applied the same amount of vaseline. The first application of IMQ or vaseline was set as day 0 for seven consecutive days. Mice in positive control group received intraperitoneal injection of MTX 1 mg/kg/day, mice in low dose and high dose triptolide groups received intraperitoneal injection of TP 0.075 mg/kg/day and 0.15 mg/kg/day, respectively. The back area of each group was photographed daily to record the skin lesions, the skin thickness was recorded using vernier calipers, and the body weight was recorded. On day 7, the mice were euthanized via CO_2_ inhalation. The mouse eyeballs were removed and centrifuged to obtain serum, the skin on the back was isolated, and the spleen was taken and photographed and weighed.

Based on the Clinical Psoriasis Area and Severity Index (PASI), a modified scoring system was developed to assess the severity of skin inflammation in mice (Van der fits et al., 2009). Scales, erythema, and thickening were scored on a scale from 0 to 4 (0 = none; 1 = slight; 2 = moderate; 3 = marked; 4 = very marked). Cumulative scales and erythema and thickening scores were used as measures of skin inflammation severity (0–12).

### 2.4 Hematoxylin and eosin staining

The dorsal skin of mice was collected on the seventh day and then immersed in 4% paraformaldehyde for 48 h. Following dehydration, the skin was encased in paraffin and sliced into 5 μm sections. The skin slides were stained with hematoxylin/eosin (H&E), observed with a microscope (Leica, Tokyo, Japan), and the images were collected.

### 2.5 Immunohistochemistry analysis

Put tissue slides in the baking oven at 65°C for 4–6 h. Paraffin was removed with xylene and ethanol. Put the slides into 0.01 M sodium citrate buffer solution and heat them in a pressure cooker for 2 min. After cooling, dropped 3%H_2_O_2_ on the slices at room temperature for 30 min and washed them with PBS. Proliferating cell nuclear antigen (PCNA) antibody or Wnt5a antibody working solution was added and incubated at 4°C for 12 h. Anti-rabbit antibody working solution was added and incubated at room temperature for 2 h. DAB solution is used as color developing agent for 15 min and rinsing them with water. The following steps were dehydration, transparency and sealing. The skin slides were observed with a microscope, and the images were collected.

### 2.6 Enzyme-linked immunosorbent assay (ELISA)

Eyeballs were extracted to obtain blood, placed at 37°C for 0.5 h, and centrifuged at 4000 rpm for 10 min at 4°C to obtain serum. IL-17A (#SEKM-0018), IL-22 (#SEKM-0023), IL-23 (#SEKM-0024), IL-6 (#SEKM-0007), and TNF-α (#SEKM-0034) in the serum were determined by ELISA kits (Solebol, Beijing, China).

### 2.7 Flow cytometry analysis of Th17 population

In order to count single cells, spleen cells are reduced to homogeneous single cells by using nylon cell strainers of 100 m length. Red blood cells are processed three times or more by using Red Blood Cell Lysis Buffer. For staining the Th17 cells, cells were stimulated with Cell Stimulation Cocktail for 6 h. Fc receptors were blocked with mouse sero block FcR before surface staining. The cells were stained with FITC-conjugated CD4 antibody for 30 min at 4°C, fixed for 30 min, and permeabilized two times. Cells were stained using PE-conjugated IL-17A antibody for 30 min at 4°C. Flowcytometer (BD Biosciences) was used for data acquisition and analysis.

### 2.8 Cell culture and treatment

HaCaT, an immortalized line of human epidermal keratinocytes, was obtained from Cell Resources Center of the Shanghai Institutes for Biological Sciences (Chinese Academy of Sciences, Shanghai, China). HaCaT cell line was cultured in Dulbecco’s modified Eagle’s medium containing 10% fetal bovine serum and 100 U/mL penicillin and 100 μg/mL streptomycin in a humidified incubator with 5% CO_2_ at 37°C. Recombinant Human IL-17A Protein was added to the cell supernatant at the concentration of 100 ng/mL to induce psoriasis-like model *in vitro*.

### 2.9 Methyl thiazolyl tetrazolium (MTT) assay

8,000 cells of HaCaT were planted into every well of 96-well plate. The cells treated with different concentrations of TP with or without the existence of 100 ng/mL of IL-17A were cultured at 37°C in 5% CO_2_. For further experiments, 25 μL MTT solution was added to cells for 4 h before the MTT test. The crystal was then dissolved in 150 μL DMSO and read at 490 nm using a microplate reader. Graphpad Prism 7.0 software was employed to determine the semi-maximum inhibition concentration (IC_50_).

### 2.10 Cell cycle analysis

TP (20, 40 or 80 nM) with or without the existence of 100 ng/mL of IL-17A were used to treat HaCaT cells for 24 h. Cells were collected and fixed within cold 70% ethanol in a dropwise fashion, and then incubated for 24 h at −20°C overnight. Ethanol was then removed and cells were stained with Propidium Iodide (0.05 mg/mL) and incubated in the dark for 20 min at RT. Cell cycles were analyzed by the flowcytometer.

### 2.11 Apoptosis assay

The ratio of apoptotic cells were analyzed using the annexin V-FITC Apoptosis Detection Kit. TP (20, 40 or 80 nM) with or without the existence of 100 ng/mL of IL-17A were used to treat HaCaT cells for 24 h. The cells were harvested and washed three times with PBS. Then, the harvested cells were stained with Annexin V-FITC and PI staining solution, according to the manufacturer’s instructions. After 30 min of incubation in the dark. The ratio of apoptotic cells were analyzed by the flowcytometer.

### 2.12 Western blot analysis

Animal tissues or cultured cells were lysed in lysis buffer and quantified. Proteins were separated on a sodium dodecyl sulfate polyacrylamide gel electrophoresis gel and transferred to polyvinylidene difluoride membrane. After blocking with 5% skimmed milk for 1.5 h at room temperature, membranes were incubated overnight with specific antibodies at 4 °C. Then, the membranes were washed three times (5 min each) with tris buffered saline tween (TBST) and required 1 h incubation at room temperature with proper secondary antibodies. After washing membranes for 5 times with TBST, immunoreactivity was detected using the electrochemiluminescence detection kit.ImageJ software was used for determination of the density of the bands.

### 2.13 Quantitative real-time PCR (qRT-PCR)

Total RNA was isolated from HaCaT cells or mouse skin tissues using TRIzol™ Reagent according to the manufacturer’s instructions. The RNA concentration and purity were then assessed using a NanoDrop microvolume spectrophotometer (Thermo). RNA was reverse transcribed to cDNA using the PrimeScriptTM RT Reagent Kit (Takara Bio, Japan). Real-time qPCR of geen expression was performed using SYBR Green and 2 × Taq PCR MasterMix and the Eppendorf Real plex 4 (Eppendorf, Hamburg, Germany). The primer sequences were as Table 1.

**TABLE 1:** Primer sequence used in this study.

	Forward	Reverse
mouse- Wnt5a	CAA​CTG​GCA​GGA​CTT​TCT​CAA	CCT​TCT​CCA​ATG​TAC​TGC​ATG​TG
mouse-GAPDH	AAT​GGA​TTT​GGA​CGC​ATT​GGT	TTT​GCA​CTG​GTA​CGT​GTT​GAT
human-Wnt5a	ATT​CTT​GGT​GGT​CGC​TAG​GTA	CGC​CTT​CTC​CGA​TGT​ACT​GC
human-CXCL 1	GAA​AGC​TTG​CCT​CAA​TCC​TG	CAC​CAG​TGA​GCT​TCC​TCC​TC
human-CXCL 2	ATT​GGT​GGC​TGT​TCC​TGA​AG	AAA​CAC​ATT​AGG​CGC​AAT​CC
human-CXCL 8	GTT​CCA​CTG​TGC​CTT​GGT​TT	GCT​TCC​ACA​TGT​CCT​CAC​AA
human-CCL 20	TGC​TGT​ACC​AAG​AGT​TTG​CTC	CCA​GTT​CTG​CTT​TGG​ATC​AGC
human-IL-1β	ATG​ATG​GCT​TAT​TAC​AGT​GGC​AA	GTC​GGA​GAT​TCG​TAG​CTG​GA
human-IL-6	ACT​CAC​CTC​TTC​AGA​ACG​AAT​TG	CCA​TCT​TTG​GAA​GGT​TCA​GGT​TG
human-IL-19	ATC​CAA​GCT​AAG​GAC​ACC​TTC​C	GTC​ACG​CAG​CAC​ACA​TCT​AAG
human-β-actin	CAT​GTA​CGT​TGC​TAT​CCA​GGC	CTC​CTT​AAT​GTC​ACG​CAC​GAT

### 2.14 Hoechst 33,342 staining

HaCaT cells were cultured on a six-well culture plate. TP (20, 40 or 80 nM) with or without the existence of 100 ng/mL of IL-17A were used to treat HaCaT cells for 24 h. 4% paraformaldelyde was used to fix HaCaT, and washed three times with PBS followed by stained with 1 mg/mL Hoechst 33,342 for 10 min in the dark. The characteristic of cell nucleus was observed by employing a fluorescence microscope (Leica, Tokyo, Japan).

### 2.15 5-ethynyl-2′-deoxyuridine (EdU) assay

The cell proliferation was measured using EdU Cell Proliferation kit. HaCaT cells were cultured on glass coverslips and treated with different concentrations of TP (20, 40 or 80 nM) and/or IL-17A for 24 h and stained with EdU-labeling mixture (10 mM) for 12 h. According to the manufacturer’s instruction, 4% paraformaldehyde was used for 10 min cell fixing at room temperature, and 0.5% TritonX-100 was used to permeabilized cells. HaCaT cells were subsequently incubated with 1 × Click-iT EdU reaction mixture in the dark for 30 min, followed by stained with Hoechst 33,342 for another 10 min. Finally, the EdU assay kit analyzed cell proliferation observed by employing a fluorescence microscope.

### 2.16 Statistical analysis

All experiments, except animal models, were conducted at least three time. GraphPad Prism 7.0 (GraphPad Software, CA, United States) was used for all statistical analyses. Data were expressed as mean ± Standard Error of Mean (SEM). Comparison of groups was subjected to one-way ANOVA (P-value <0.05 was considered statistically significant).

## 3 Results

### 3.1 TP alleviated IMQ-induced psoriasis-like skin lesions in mice

**FIGURE 1:**
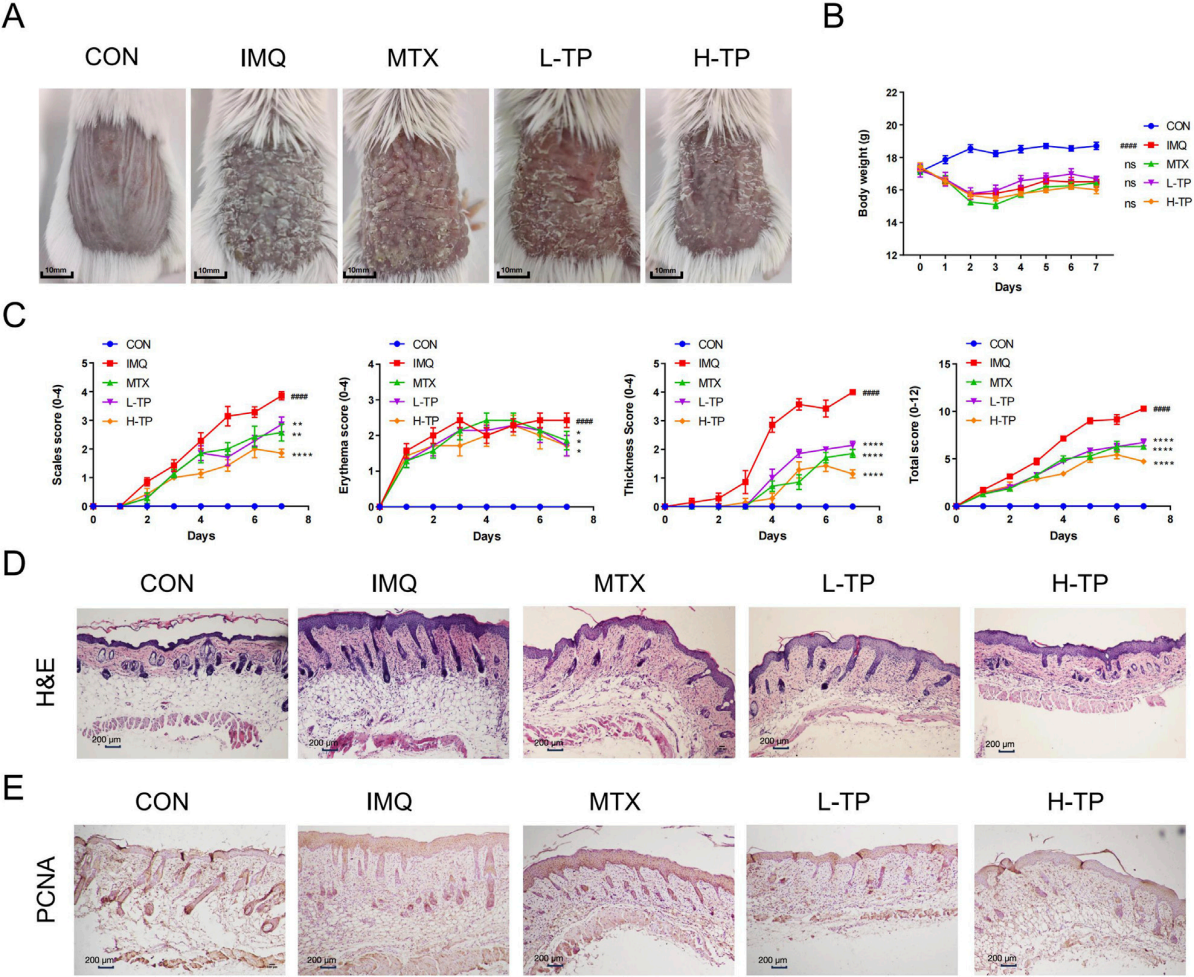
TP alleviated the IMQ-induced psoriasis-like mouse skin lesion. **(A)** Macroscopic presentation of mouse back skin after 7 days of treatment. **(B)** Daily body weight of mice (n = 7). **(C)** The scales, erythema and thickness of the back skin were evaluated daily from the first day according to the Psoriasis Area Severity Index (PASI), with a score ranging from 0 to 4, and the total score was calculated: scales + erythema + thickness (n = 7). **(D)** Haematoxylin and eosin (H&E) staining of back skin lesions from different groups of mice (scale bar = 200 μm, n ≥ 3). **(E)** Immunohistochemical analysis of PCNA in the epidermis (scale bar = 200 μm, n ≥ 3). All Data are represented as the mean ± SEM. ^*####*^ *p* < 0.0001 versus CON; *p < 0.05, **p < 0.01, ****p < 0.001,****p < 0.0001 versus IMQ; ns, not significant. IMQ, imiquimod; MTX, methotrexate 1 mg/kg; L-TP, triptolide 75 μg/kg; H-TP, triptolide 150 μg/kg.

We investigated the therapeutic effect of TP on the psoriasis-like skin inflammation model by applying IMQ on the shaved back of mice daily to induce psoriasis. The results showed that symptoms such as scale formation, erythema, and skin thickness decreased significantly in TP-treated mice compared with those in IMQ-treated mice (Figure 1A). Furthermore, the therapeutic effect of TP was dose-dependent, as well as this effect on psoriasis-like symptoms was similar to that of MTX (Figure 1A). Additionally, TP-treated mice showed significant body weight loss compared with control mice; however, no significant difference was observed between the body weights of TP-treated and IMQ-treated mice (Figure 1B). This result was found by calculating the PASI scores of each group of mice (Figure 1C). Histopathological analysis and PCNA staining of skin tissues showed that TP treatment significantly inhibited IMQ-induced epidermal thickening (Figure 1D). Given the observation of abundant inflammatory cells exhibiting macrophage-like nuclear morphology, we performed immunofluorescence staining for the macrophage-specific marker F4/80. TP treatment induced a dose-dependent attenuation of dermal inflammatory cell infiltration, with macrophage density being significantly reduced compared to the IMQ-induced group (Supplementary Figure S3). The number of PCNA-positive proliferating keratinocytes in the skin of TP-treated mice was significantly lower than that in IMQ-treated mice (Figure 1E).

### 3.2 TP exerted an anti-inflammatory effect on the psoriasis model

**FIGURE 2:**
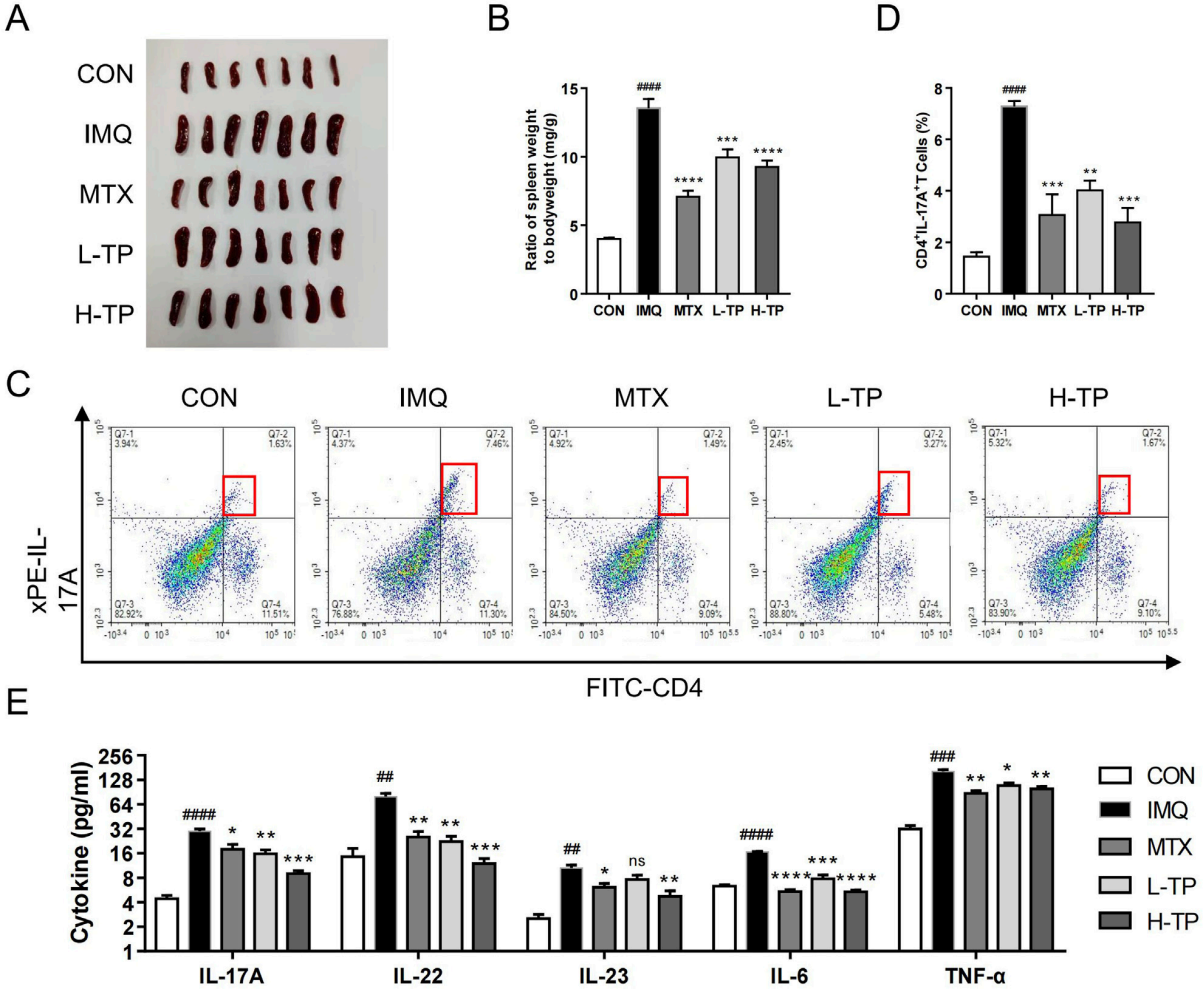
TP shows significant anti-inflammatory effect on psoriasis *in vivo*. **(A)** Macroscopic presentation of spleens of mice in each group. **(B)** Spleen index (spleen weight/body weight) was calculated (n = 7). **(C)** The frequency of Th17 cells was in spleen of each group analyzed by flow cytometry (n ≥ 3). **(D)** Statistical plots of Th17 cell frequency in spleen of each group (n ≥ 3). **(E)** The protein expression of IL-17A, IL-22, IL-23, IL-6 and TNF-α in serum of each group were analyzed by ELISA (n ≥ 3). All Data are represented as the mean ± SEM. ^*##*^ *p* < 0.01, ^*###*^ *p* < 0.001, ^*####*^ *p* < 0.0001 versus CON; *p < 0.05, **p < 0.01, ***p < 0.001, ****p < 0.0001 versus IMQ; ns, not significant.

Spleen enlargement was inhibited as well as the spleen index (the ratio of spleen weight to body weight) decreased after TP treatment in psoriasis model mice, which suggested that the anti-inflammatory effects of MTX and TP were similar (Figures 2A, B). Th17 cells play an important role in psoriasis pathogenesis; thus, we performed flow cytometry to detect Th17 cells in the mouse spleen and found that the Th17 cell proportion decreased in the spleen after TP treatment (Figures 2C, D). To further explore the inflammatory cytokines regulated by TP treatment, we extracted serum from the mice and analyzed the expression of inflammatory genes, such as IL-17A, IL-22, IL-23, TNF-α, and IL-6, which were closely related to psoriasis pathogenesis. ELISA experiments showed that *in vivo* serum levels of inflammatory cytokines decreased significantly in TP-treated mice, and these levels were lower in the H-TP group mice than in the L-TP group mice (Figure 2E).

### 3.3 IMQ-upregulated Wnt5a expression was inhibited by TP

**FIGURE 3:**
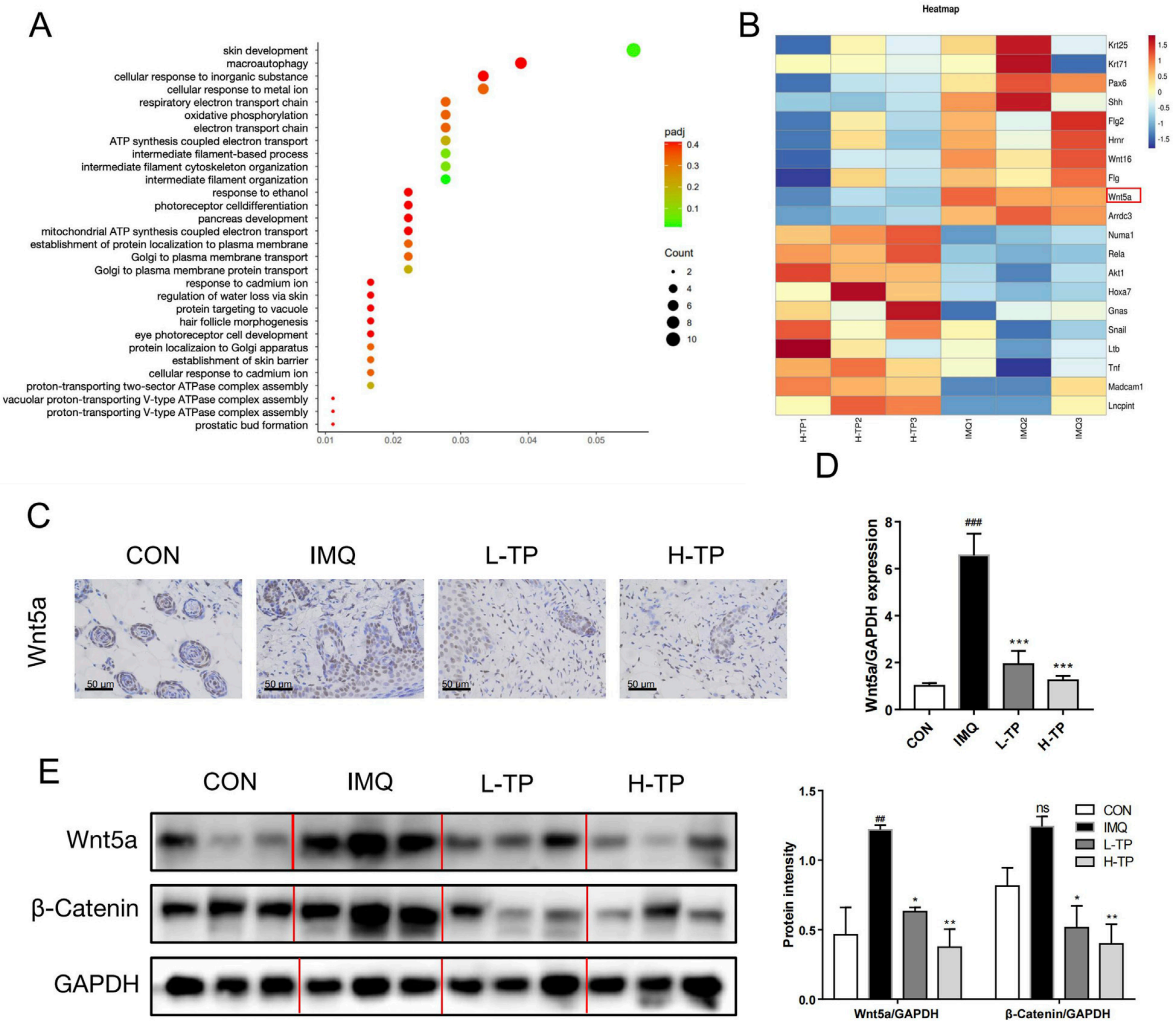
Increased Wnt5a expression in IMQ-induced psoriasis mice was inhibited by TP treatment **(A)** GO enrichment analysis of back skin of mice in the IMQ group and H-TP group (n = 3). **(B)** Heat maps showing differentially expressed genes in the classification of skin development in the back skin of IMQ and H-TP groups mice (n = 3). Increased transcript levels are colored red and decreased levels are colored green. **(C)**The expression of Wnt5a was evaluated by immunohistochemical staining of mouse back skin (Scale bar = 50 μm, n = 3). **(D)** Total RNA was isolated from back skin tissues, and RT-PCR was used to investigate the mRNA levels of Wnt5a (n = 3). **(E)** The expression of Wnt5a and β-Catenin in the back skin of different groups were measured with Western blot. The column figures of quantitative Wnt5a and β-Catenin protein expression data are shown on the right (n = 3). All Data are represented as the mean ± SEM. ^*##*^ *p* < 0.01, ^*###*^ *p* < 0.001 versus CON; *p < 0.05, **p < 0.01, ***p < 0.001 versus IMQ; ns, not significant.

To elucidate the mechanism underlying the anti-psoriasis effect of TP, we analyzed and compared the transcriptome data of the skin lesion tissues of IMQ- and H-TP-treated mice by RNA sequencing. Gene Ontology (GO) molecular function analysis showed that TP treatment significantly affected skin development in the mice (Figure 3A) and downregulated the expression of Wnt5a (Figure 3B), which is highly expressed in the skin lesions of patients with psoriasis. By performing immunohistochemistry, qRT-PCR, and Western blotting of the mouse skin lesions, we showed that IMQ treatment increased Wnt5a mRNA and protein levels in the lesions, whereas TP treatment significantly inhibited IMQ-induced Wnt5a overexpression (Figures 3C–E). However, MTX treatment failed to attenuate IMQ-induced Wnt5a overexpression in the experimental model (Supplementary Figure S4).

### 3.4 TP inhibited IL-17a-induced upregulated Wnt5a expression in HaCaT cells

**FIGURE 4:**
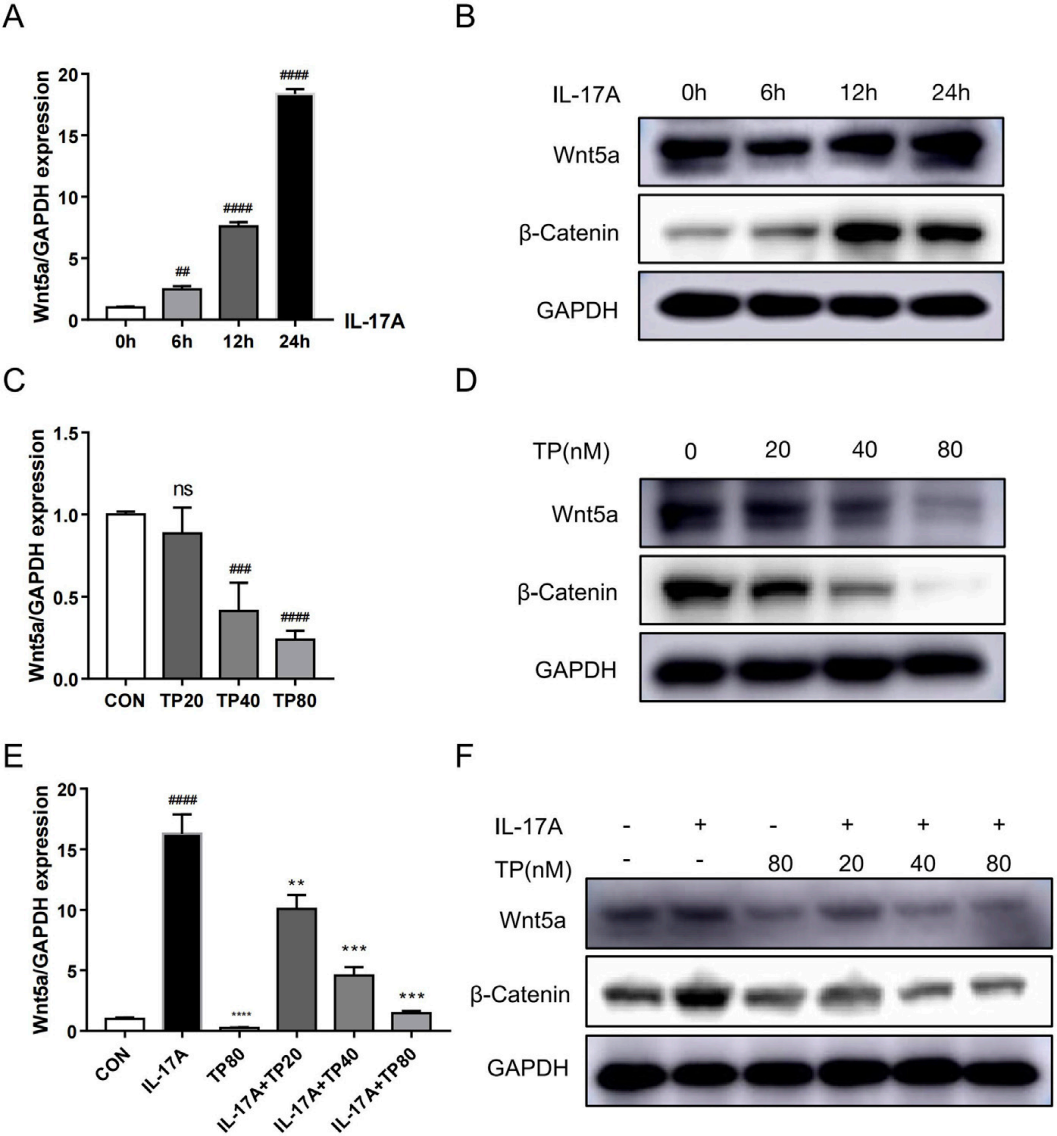
TP suppresses IL-17A-induced upregulation of the Wnt5a/β-catenin signaling pathway in HaCaT cells. **(A)** The mRNA levels of Wnt5a induced by IL-17A in HaCaT cells were analyzed by qRT-PCR. **(B)** The Wnt5a and β-Catenin protein levels induced by IL-17A in HaCaT cells were detected by Western blot. **(C)** The mRNA levels of Wnt5a by TP-treated in HaCaT cells were analyzed by qRT-PCR. **(D)** The Wnt5a and β-Catenin protein levels by TP-treated in HaCaT cells were detected by Western blot. **(E)** The mRNA levels of Wnt5a treated by IL-17A and/or TP in HaCaT cells were analyzed by qRT-PCR. **(F)** The Wnt5a and β-Catenin protein levels treated by IL-17A and/or TP in HaCaT cells were detected by Western blot. All Data are represented as the mean ± SEM, n = 3. ^*##*^ *p* < 0.01, ^*###*^ *p* < 0.001, ^*####*^ *p* < 0.0001 versus CON or 0h; **p < 0.01, ***p < 0.001, ****p < 0.0001 versus IL-17A; ns, not significant. IL-17A, interleukin-17A 100 ng/mL; TP20, triptolide 20 nM; TP40, triptolide 40 nM; TP80, triptolide 80 nM.

To evaluate our conjecture that TP can regulate Wnt5a at the cellular level, we determined mRNA and protein levels in HaCaT cells treated with different concentrations of TP by performing qRT-PCR and Western blotting. The TP concentration was determined based on the IC50 value calculated by performing the MTT assay (Supplementary Figure S1). Although 20 nM TP had no significant effect on Wnt5a expression, 40 and 80 nM TP significantly suppressed Wnt5a expression in HaCaT cells (Figures 4C, D). Additionally, Western blotting was performed to examine the level of β-catenin, an important gene downstream of Wnt5a, in HaCaT cells, and the results showed that TP significantly downregulated Wnt5a/β-catenin signaling in a dose-dependent manner (Figure 4D; Supplementary Figure S5B). Fibroblast-like synoviocytes treated with IL-17A showed upregulated Wnt5A mRNA expression (Lavocat et al., 2016). When we treated HaCaT cells with 100 ng/mL IL-17A, Wnt5a mRNA levels increased significantly (Figure 4A), and they continued to increase with increasing treatment time within 24 h (Figure 4A). The Western blotting results confirmed that IL-17A treatment increased Wnt5a and β-catenin protein levels in HaCaT cells (Figure 4A; Supplementary Figure S5C). Moreover, in HaCaT cells treated with different concentrations of TP with or without 100 ng/mL IL-17A, Wnt5a expression was successfully inhibited by TP and was not upregulated by IL-17A. Similarly, β-catenin expression was regulated by IL-17A and TP (Figures 4E, F; Supplementary Figure S5C).

### 3.5 TP inhibited HaCaT cell hyperproliferation induced by IL-17A

**FIGURE 5:**
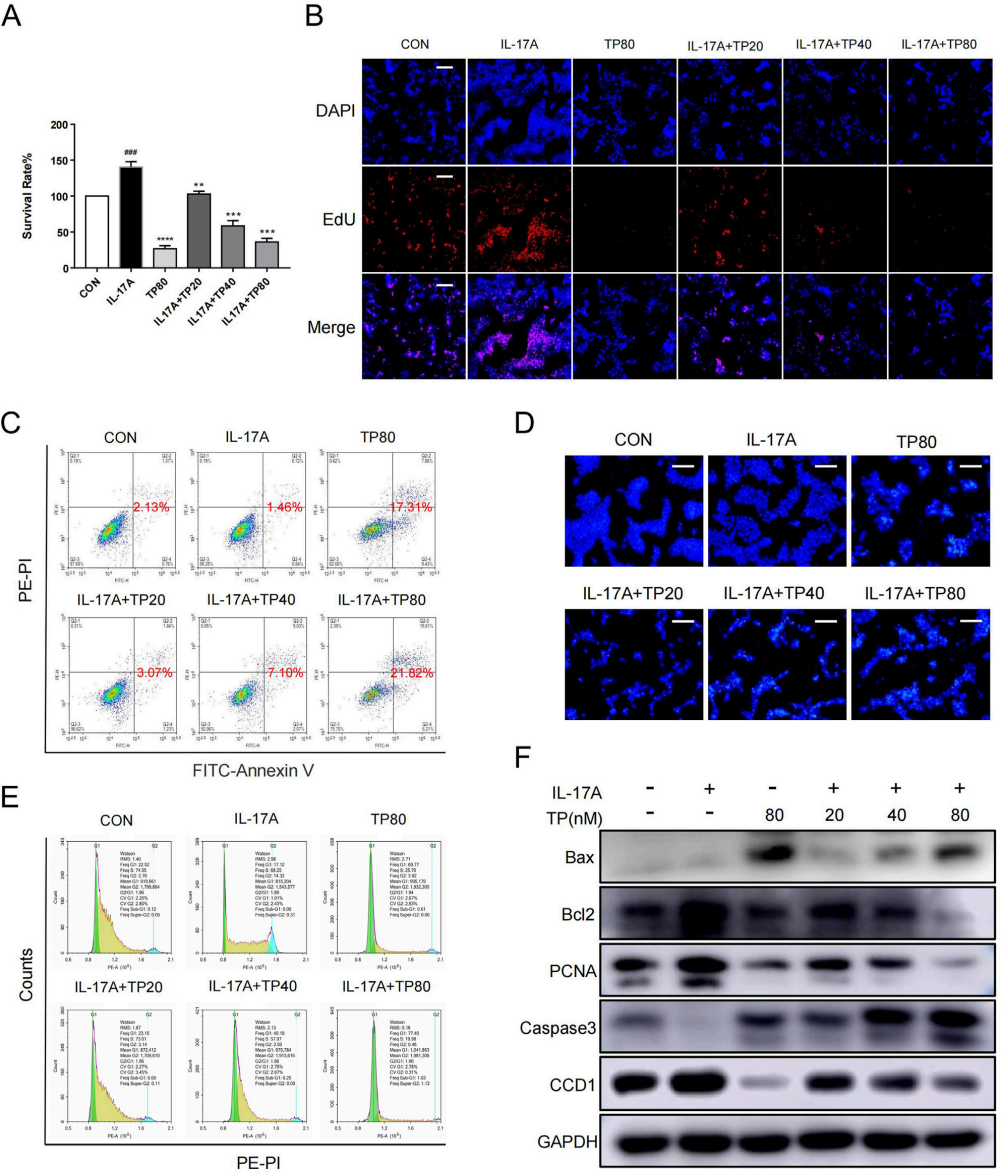
TP effectively inhibited the proliferation, promoted the apoptosis and arrested the cell cycle of HaCaT cells that stimulated by IL-17A **(A)** After HaCaT cells were treated with IL-17A and/or TP for 24 h, the proliferation of HaCaT cells was detected by MTT assay. **(B)** 5-ethynyl20-deoxyuridine (EdU) staining assayed the number of proliferating HaCaT cells that had been treated with IL-17A and/or TP for 24 h. Positive cells were stained by EdU (red) and total cell nucleus was stained with Hoechst (blue) (scale bar = 100 μm, n = 3). **(C)** Representative scatter plots of the flow cytometry analysis of the fraction of apoptotic HaCaT cells that had been treated with IL-17A and/or TP for 24 h. **(D)** The apoptotic characteristics in HaCaT cell nucleus presented by Hoechst Staining assay. After treated with IL-17A and/or TP for 24 h before stained with Hoechst 33,342 staining, the nucleus was observed and photographed (scale bar = 100 μm, n = 3). **(E)** The analysis of cell cycle phase of HaCaT cells treated with IL-17A and/or TP for 24 h. Cells were stained with propidium iodide (PI) and determined with Flow cytometry. **(F)** After HaCaT cells were treated with IL-17A and/or TP for 24 h, Western blot analysis showed the levels of proteins involved apoptosis, proliferation and cell cycle. All Data are represented as the mean ± SEM, n = 3. ^*###*^ *p* < 0.001 versus CON; **p < 0.01, ***p < 0.001, ****p < 0.0001 versus IL-17A.

HaCaT cell stimulation with IL-17A induced hyperproliferation (Di Cesare et al., 2009; Kim and Krueger, 2017). When 100 ng/mL IL-17A induced HaCaT cell hyperproliferation, their treatment with TP (20 nM, 40 nM, and 80 nM) led to decreased cell survival (Figure 5A). The EdU assay showed that the proportion of HaCaT cells to be proliferated was significantly more in the IL-17A treatment group than in the TP treatment group, and the lower proportion in the latter group was independent of IL-17A treatment (Figure 5B; Supplementary Figure S2A). Additionally, TP treatment significantly promoted HaCaT cell apoptosis (Figures 5C, D; Supplementary Figures S2B, C). The cell cycle analysis showed that TP treatment alone significantly increased the number of cells in the G1 phase compared with those in the control group (Figure 5E; Supplementary Figure S2D). Compared with the IL-17A group, IL-17A and TP co-treatment significantly increased the number of cells in the G1 phase in a dose-dependent manner (Figure 5E; Supplementary Figure S2D). Subsequently, we performed Western blotting to investigate the levels of proliferation-, apoptosis-, and cell-cycle-related proteins. TP significantly increased the levels of caspase three and Bax and decreased the level of bcl-2, thereby confirming the occurrence of apoptosis (Figure 5F). IL-17A increased PCNA expression, which was inhibited by TP, thereby suggesting the involvement of TP in suppressing keratinocyte hyperproliferation in psoriasis. The effect of TP on cell cycle arrest in HaCaT cells was attributed to CCD1 expression inhibition. Thus, TP inhibited IL-17A-induced keratinocyte hyperproliferation.

### 3.6 TP inhibited the IL-17A-induced inflammatory response in HaCaT cells

**FIGURE 6:**
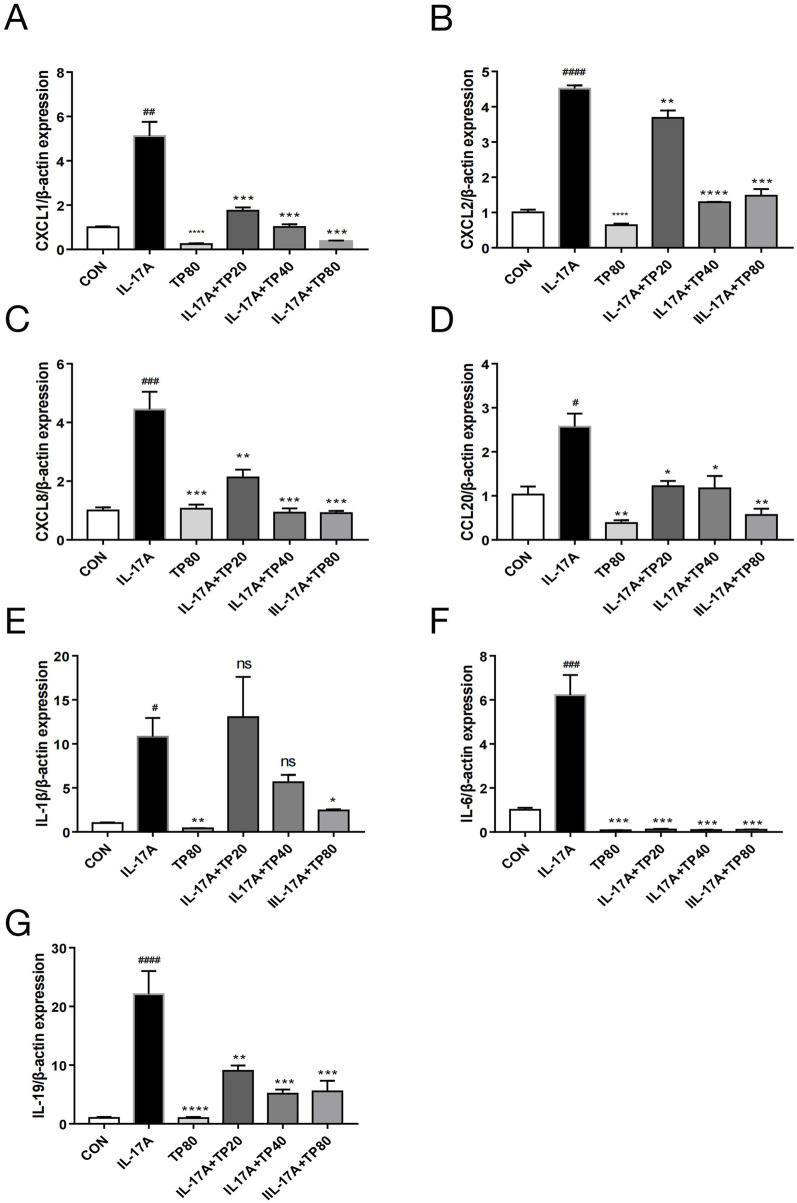
TP affected the expression of chemokines and cytokines in HACAT cells stimulated with IL-17A. After HaCaT cells were treated with IL-17A and/or TP for 24 h, the mRNA levels of chemokines and cytokines were determined by qPCR. **(A)** CXCL1; **(B)** CXCL2; **(C)** CXCL8; **(D)** CCL20; **(E)** IL-1β; **(F)** IL-6; **(G)** IL-19. All Data are represented as the mean ± SEM, n = 3. ^*#*^ *p* < 0.05, ^*##*^ *p* < 0.01, ^*###*^ *p* < 0.001, ^*####*^ *p* < 0.0001 versus CON; *p < 0.05, **p < 0.01, ***p < 0.001, ****p < 0.0001 versus IL-17A; ns, not significant.

IL-17A elicits an amplified inflammatory response in keratinocyte (Di Cesare et al., 2009; Kim and Krueger, 2017; Mease, 2015). The mRNA levels of several cytokines, including CXCL1, CXCL2, CXCL8, CCL20, IL-1β, IL-6, and IL-19, in HaCaT cells increased significantly after exogenous IL-17A addition (Figure 6). However, compared with IL-17A treatment, TP monotherapy significantly reduced the mRNA levels of CXCL1, CXCL2, CCL20, IL-1β, and IL-6, whereas no significant effect was observed on the levels of CXCL8 and IL-19. However, the levels of these cytokines decreased significantly after TP and IL-17A co-treatment, which suggested that TP treatment inhibited the inflammatory response triggered by IL-17A in keratinocytes.

## 4 Discussion

TP, a diterpenoid, has been shown to demonstrate potent immunosuppressive and anti-inflammatory effects (Brinker et al., 2007), and has been proposed as a possible treatment option for psoriasis.However, current reports on the therapeutic application of TP in psoriasis are scarce, and the mechanisms underlying its effects remain unelucidated. In this study, we characterized the biological role of TP in psoriasis *in vivo* and *in vitro*. Treatment with TP demonstrated efficacy in alleviating IMQ-induced psoriasis-like skin lesions in mice and inhibiting HaCaT cell hyperproliferation induced by IL-17A. Mechanically, TP inhibits Wnt5a/β-catenin signaling, which may be a potential mechanism by which TP improves psoriasis.

Psoriasis is a prevalent, chronic skin condition that is orchestrated by both the innate and adaptive immune responses, with key contributions from keratinocytes, dendritic cells, and T cells. IMQ, an agonist of toll-like receptor (TLR)7 and TLR8, is widely used to induce a mouse model of psoriasis (Jabeen et al., 2020). Herein, IMQ-treated mice mimicked the symptoms of human psoriasis such as scale formation, erythema, and thickened skin. In terms of immune inflammation, treatment with IMQ resulted in spleen enlargement, the number of Th17 cells increased significantly in the spleen, and the infiltration of inflammatory cells and cytokines such as IL-23 and IL-17A increased in the skin (Flutter and Nestle, 2013; Van der fits et al., 2009). Animal experiments have demonstrated that TP suppressed scaling, erythema, and thickening in the psoriasis model and reduced skin thickness and immune cell infiltration in the back tissues of psoriasis model mice. Moreover, TP exerted its therapeutic effects on the spleen, blood circulation system, and skin tissues, and finally exerted systemic therapeutic effects on psoriasis in mice. Thus, TP exerted a regulatory effect on the IL23/Th17 axis and an inhibitory effect on IL-17A production and secretion during psoriasis treatment.

Nardi et al. reported that Triptolide inhibits Wnt signaling in NSCLC (Nardi et al., 2018), there is no report on its mechanism of action in the treatment of psoriasis. To elucidate the mechanism of action of TP, RNA sequencing was performed using the skin lesion tissue from the mice in the IMQ and H-TP groups. Herein, the IMQ-induced psoriasis model mice showed a similar increase in Wnt5a and β-catenin mRNA expression and protein levels and Wnt5a mRNA expression in skin development classification was downregulated by TP treatment.

Johann E. Gudjonsson et al. treated keratinocytes with TGF-α, TNF-α, IFN-γ, or IL-1α to upregulate Wnt5a expression; however, no stimulatory effect of IL-17A on Wnt5a expression was found (Romanowska et al., 2009). Additionally, the stimulatory effect of IL-17A on Wnt5a was observed in fibroblast-like synoviocytes (Lavocat et al., 2016). Moreover, TP downregulated the Wnt5a/β-catenin pathway in HaCaT cells with or without IL-17A treatment. Therefore, we hypothesize that TP plays a regulatory role in HaCaT cells by antagonizing IL-17A-induced Wnt5a/β-catenin pathway activation.

Wnt5a regulates various cellular functions including cell proliferation and inflammation, and Wnt5a knockdown downregulates PCNA and CCD1 expression (Zhang et al., 2015). PCNA is used to examine epidermal hyperproliferation in psoriasis and the potential for malignancy in skin lesions, and CCD1 is a key regulator of cell cycle progression in psoriasis, and both are downstream of the Wnt5a/β-catenin pathway genes (Castilla et al., 2012). The inhibitory effect of TP on IL-17A-induced HaCaT proliferation was determined. Subsequently, the levels of the proliferation markers PCNA and bcsl2 were examined, which reflected the pro-proliferation effect of IL-17A and the anti-apoptotic effect of TP on HaCaT cells. Similarly, a previous study showed that TP treatment inhibited IL-22-stimulated keratinocyte proliferation by upregulating miR-181b-5p (He et al., 2020). Psoriatic keratinocytes show enhanced resistance to apoptosis, which may be one of the key factors in psoriasis pathogenesis (Chimenti et al., 2018). TP can exert an apoptosis-inducing effect in various cells (Mohammad et al., 2015). Herein, TP induced apoptosis in HaCaT cells, which was independent of the presence of IL-17A as evidenced by the levels of the apoptotic markers Bax and caspase 3. Wnt5a increased the proportion of HaCaT cells arrested at the G2/M phase, whereas it decreased the proportion of HaCaT cells arrested at the G0/G1 phase by increasing CCD1 levels (Wang et al., 2018). This is consistent with our findings that Wnt5a expression was regulated by IL-17A or TP in HaCaT cells.

IL-17A induced keratinocyte proliferation, produced pro-inflammatory cytokines (including IL-1b, IL-6, and IL-19), and formed a positive feedback loop to maintain inflammation. Furthermore, research has found that keratinocytes can produce multiple chemokines, including CXCL1, CXCL2, CXCL8 and CCL20, under the stimulation of IL-17A (Flutter and Nestle, 2013). The generation of these chemokines further intensifies the inflammatory response. The present study showed that TP treatment inhibited the mRNA expression of proinflammatory cytokines and chemokines in HaCaT cells, and this inhibitory effect was almost independent of the presence of exogenous IL-17A. Additionally, endothelial cell proliferation and inflammation play an important role in psoriasis development (Niu et al., 2022). Wnt5a activates the NF-κB signaling pathway in endothelial cells via the Wnt5a/Ca^2+^/protein kinase C pathway, thereby increasing the levels of cytokines including IL-8, IL-6, and IL-1α in endothelial cells (Kim et al., 2010). TP can inhibit HUVEC proliferation, migration, and angiogenesis (Jia et al., 2022). While previous studies have highlighted TP’s suppression of IL-17A via Th17 inhibition or NF-κB signaling in conditions such as liver fibrosis and rheumatoid arthritis (Horitani and Shiojima, 2024; Jiang et al., 2025), our work uncovers a previously unrecognized mechanism: TP’s regulation of the Wnt5a/β-catenin axis in keratinocytes. This pathway bridges IL-17A-driven inflammation to epidermal hyperplasia, a hallmark of psoriasis. In this regard, Wnt5a/β-catenin pathway regulation can inhibit the stimulatory effect of IL-17A on HaCaT. However, the extent to which this stimulatory effect depends on the Wnt5a/β-catenin pathway remains unclear.

## 5 Conclusion

**FIGURE 7:**
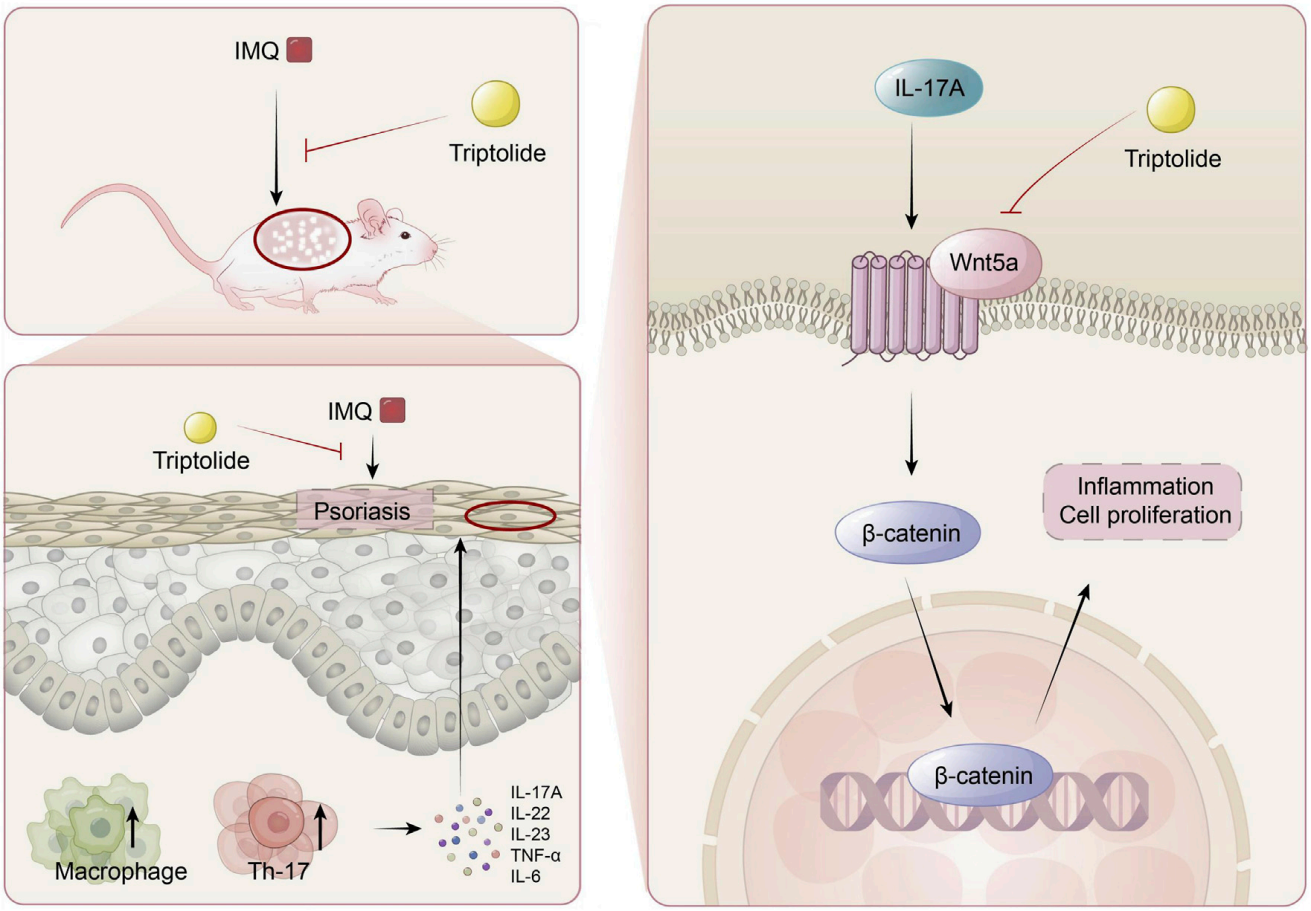
The mechanism of TP in the treatment of psoriasis. TP can effectively alleviate imiquimod-induced psoriasis symptoms in mice, such as epidermal thickening, Th17 cell differentiation, macrophage activation, and the release of inflammatory factors like IL-17A in serum. In keratinocytes, TP targets the Wnt5A/β-catenin signaling pathway to suppress the excessive proliferation of keratinocytes.

In conclusion, we demonstrated that TP treatment alleviated IMQ-induced psoriasis-like symptoms in a mouse model and inhibited IL-17A-induced proliferation and inflammation in HaCaT cells (Figure 7). Owing to IMQ-induced psoriasis, Wnt5a expression was upregulated in mice similar to in patients with psoriasis. Treatment with TP was accompanied by Wnt5a/β-catenin signaling inhibition. IL-17A was a crucial factor associated with abnormally upregulated Wnt5a expression during psoriasis development. Therefore, we propose that TP suppresses keratinocyte hyperproliferation and inflammatory responses in psoriasis by regulating the Wnt5a/β-catenin signaling pathway.

## Data Availability

The original contributions presented in the study are included in the article/Supplementary Material, further inquiries can be directed to the corresponding authors.

## Abbreviations

Bax, bcl-2 associated X protein; Bcl2, b-cell lymphoma-2; CCL20, chemokine ligand 20; CXCL1, chemokine ligand 1; DMSO, dimethyl sulfoxide; EDTA, ethylene diamine tetraacetic acid; ELISA, enzyme-linked immunosorbent assay; FITC, fluorescein isothiocyanate; GAPDH, glyceraldehyde 3-phosphate dehydrogenase; GO, gene ontology; HaCaT, human immortalized keratinocytes; HE, hematoxylin-eosin; IC_50_, the half maximal inhibitory concentrations; IHC, immunohistochemistry; IL17A, interleukin 17; IMQ, imiquimod; MTT, 3-(4,5- dimethylthiazol-2-yl)-2,5-diphenyltetrazolium bromide; MTX, methotrexate; PASI, psoriasis area and severity index; PCNA, phosphate balanced solution; TBST, tris buffered saline tween; TEMED, tetramethylethylenediamine; Th17, T helper cell 17; TLR7, toll-like receptors 7; TNF-α, tumor necrosis factor-α; TP, triptolide; Wnt5a, winglesstype MMTV integration site family member 5a.

## References

[B1] Bian G. Wang L. Xie Q. Wang Y. Feng H. Yu Y. (2021). Dgt, a novel heterocyclic diterpenoid, effectively suppresses psoriasis via inhibition of Stat3 phosphorylation. Br. J. Pharmacol. 178 (3), 636–653. 10.1111/bph.15306 33140855

[B2] Brinker A. M. Ma J. Lipsky P. E. Raskin I. (2007). Medicinal chemistry and pharmacology of genus Tripterygium (celastraceae). Phytochemistry 68 (6), 732–766. 10.1016/j.phytochem.2006.11.029 17250858 PMC3867260

[B3] Bugaut H. Aractingi S. (2021). Major role of the il17/23 Axis in psoriasis supports the development of new targeted therapies. Front. Immunol. 12, 621956. 10.3389/fimmu.2021.621956 33717124 PMC7948519

[B4] Castilla C. Mcdonough P. Tumer G. Lambert P. C. Lambert W. C. (2012). Sometimes it takes darkness to see the light: pitfalls in the interpretation of cell proliferation markers (Ki-67 and pcna). SKINmed 10 (2), 90–92. 22545323

[B5] Chen S. R. Dai Y. Zhao J. Lin L. Wang Y. Wang Y. (2018). A mechanistic overview of triptolide and celastrol, natural products from Tripterygium wilfordii hook F. Front. Pharmacol. 9, 104. 10.3389/fphar.2018.00104 29491837 PMC5817256

[B6] Chimenti M. S. Sunzini F. Fiorucci L. Botti E. Fonti G. L. Conigliaro P. (2018). Potential role of cytochrome C and tryptase in psoriasis and psoriatic arthritis pathogenesis: focus on resistance to apoptosis and oxidative stress. Front. Immunol. 9, 2363. 10.3389/fimmu.2018.02363 30429845 PMC6220124

[B7] Di Cesare A. Di Meglio P. Nestle F. O. (2009). The il-23/Th17 Axis in the immunopathogenesis of psoriasis. J. Invest Dermatol 129 (6), 1339–1350. 10.1038/jid.2009.59 19322214

[B8] Doherty S. D. Van Voorhees A. Lebwohl M. G. Korman N. J. Young M. S. Hsu S. (2008). National psoriasis foundation consensus statement on screening for latent tuberculosis infection in patients with psoriasis treated with systemic and biologic agents. J. Am. Acad. Dermatol 59 (2), 209–217. 10.1016/j.jaad.2008.03.023 18485527

[B9] Dou J. Zhang L. Xie X. Ye L. Yang C. Wen L. (2017). Integrative analyses reveal biological pathways and key genes in psoriasis. Br. J. Dermatol 177 (5), 1349–1357. 10.1111/bjd.15682 28542811

[B10] Ferrara F. Verduci C. Laconi E. Mangione A. Dondi C. Del Vecchio M. (2024). Current therapeutic overview and future perspectives regarding the treatment of psoriasis. Int. Immunopharmacol. 143 (Pt 1), 113388. 10.1016/j.intimp.2024.113388 39405929

[B11] Flutter B. Nestle F. O. (2013). Tlrs to cytokines: mechanistic insights from the imiquimod mouse model of psoriasis. Eur. J. Immunol. 43 (12), 3138–3146. 10.1002/eji.201343801 24254490

[B12] Galluzzo M. D'Adamio S. Bianchi L. Talamonti M. (2016). Brodalumab for the treatment of psoriasis. Expert Rev. Clin. Immunol. 12 (12), 1255–1271. 10.1080/1744666X.2016.1246957 27718760

[B13] Garzorz-Stark N. Eyerich K. (2019). Psoriasis pathogenesis: keratinocytes are back in the spotlight. J. Invest Dermatol 139 (5), 995–996. 10.1016/j.jid.2019.01.026 31010530

[B14] Hawkes J. E. Yan B. Y. Chan T. C. Krueger J. G. (2018). Discovery of the il-23/il-17 signaling pathway and the treatment of psoriasis. J. Immunol. 201 (6), 1605–1613. 10.4049/jimmunol.1800013 30181299 PMC6129988

[B15] He Q. Zhang B. Hu F. Long J. Shi Q. Pi X. (2020). Triptolide inhibits the proliferation of hacat cells induced by Il22 via upregulating mir-181b-5p. Drug Des. Devel Ther. 14, 2927–2935. 10.2147/DDDT.S254466 PMC7383028 32801634

[B16] Horitani K. Shiojima I. (2024). Wnt signaling in cardiac development and heart diseases. vitro Cell. and Dev. Biol. Animal 60 (5), 482–488. 10.1007/s11626-024-00917-z PMC11126472 38709417

[B17] Huang T. H. Lin C. F. Alalaiwe A. Yang S. C. Fang J. Y. (2019). Apoptotic or antiproliferative activity of natural products against keratinocytes for the treatment of psoriasis. Int. J. Mol. Sci. 20 (10), 2558. 10.3390/ijms20102558 31137673 PMC6566887

[B18] Jabeen M. Boisgard A. S. Danoy A. El Kholti N. Salvi J. P. Boulieu R. (2020). Advanced characterization of imiquimod-induced psoriasis-like mouse model. Pharmaceutics 12 (9), 789. 10.3390/pharmaceutics12090789 32825447 PMC7558091

[B19] Jia L. Zhu S. Zhu M. Huang L. Xu S. Luo Y. (2022). Triptolide inhibits the biological processes of huvecs and Hepg2 cells via the serine palmitoyltransferase long chain base subunit 2/sphingosine-1-phosphate signaling pathway. Dis. Markers 2022, 9119423. 10.1155/2022/9119423 36438896 PMC9699786

[B20] Jiang S. Jiang Y. Feng J. Hou J. Qin Z. Wang Y. (2025). Triptolide combined with salvianolic acid B alleviates ccl 4 -induced liver fibrosis by suppressing the Th17/Il-17a Axis. Int. Immunopharmacol. 150, 114300. 10.1016/j.intimp.2025.114300 39965387

[B21] Kim J. Kim J. Kim D. W. Ha Y. Ihm M. H. Kim H. (2010). Wnt5a induces endothelial inflammation via beta-catenin-independent signaling. J. Immunol. 185 (2), 1274–1282. 10.4049/jimmunol.1000181 20554957

[B22] Kim J. Krueger J. G. (2017). Highly effective new treatments for psoriasis target the Il-23/Type 17 T cell Autoimmune Axis. Annu. Rev. Med. 68, 255–269. 10.1146/annurev-med-042915-103905 27686018

[B23] Kim J. E. Hyun Bang S. Choi J.Ho Kim C. D. Won C. H. Lee M. W. (2016). Interaction of Wnt5a with Notch1 is Critical for the pathogenesis of psoriasis. Ann. Dermatology 28 (1), 45–54. 10.5021/ad.2016.28.1.45 PMC4737835 26848218

[B24] Lavocat F. Osta B. Miossec P. (2016). Increased sensitivity of rheumatoid synoviocytes to schnurri-3 expression in Tnf-Alpha and Il-17a induced osteoblastic differentiation. Bone 87, 89–96. 10.1016/j.bone.2016.04.008 27072520

[B25] Mease P. J. (2015). Inhibition of interleukin-17, interleukin-23 and the Th17 cell pathway in the treatment of psoriatic arthritis and psoriasis. Curr. Opin. Rheumatol. 27 (2), 127–133. 10.1097/BOR.0000000000000147 25599143

[B26] Mohammad R. M. Muqbil I. Lowe L. Yedjou C. Hsu H. Y. Lin L. T. (2015). Broad targeting of resistance to apoptosis in cancer. Semin. Cancer Biol. 35 (Suppl. l (0), S78–S103. 10.1016/j.semcancer.2015.03.001 25936818 PMC4720504

[B27] Nardi I. Reno T. Yun X. W. Sztain T. Wang J. Dai H. (2018). Triptolide inhibits Wnt signaling in nsclc through upregulation of multiple Wnt inhibitory factors via epigenetic modifications to histone H3. Int. J. Cancer 143 (10), 2470–2478. 10.1002/ijc.31756 30006924 PMC6483070

[B28] Niu X. Han Q. Li X. Li J. Liu Y. Li Y. (2022). EDIL3 influenced the αvβ3-FAK/MEK/ERK axis of endothelial cells in psoriasis. J. Cell Mol. Med. 26 (20), 5202–5212. 10.1111/jcmm.17544 36065978 PMC9575107

[B29] Pashirzad M. Shafiee M. Rahmani F. Behnam-Rassouli R. Hoseinkhani F. Ryzhikov M. (2017). Role of Wnt5a in the pathogenesis of inflammatory diseases. J. Cell Physiol. 232 (7), 1611–1616. 10.1002/jcp.25687 27859213

[B30] Pender E. K. Kirby B. (2024). An update on topical therapies for psoriasis. Curr. Opin. rheumatology 36 (4), 289–294. 10.1097/bor.0000000000001018 38651512

[B31] Reischl J. Schwenke S. Beekman J. M. Mrowietz U. Sturzebecher S. Heubach J. F. (2007). Increased expression of Wnt5a in psoriatic plaques. J. Invest Dermatol 127 (1), 163–169. 10.1038/sj.jid.5700488 16858420

[B32] Romanowska M. Evans A. Kellock D. Bray S. E. McLean K. Donandt S. (2009). Wnt5a exhibits Layer-specific expression in Adult skin, is upregulated in psoriasis, and Synergizes with Type 1 interferon. Plos One 4 (4), e5354. 10.1371/journal.pone.0005354 19399181 PMC2670517

[B33] Tian F. Mauro T. M. Li Z. (2019). The Pathological role of Wnt5a in psoriasis and psoriatic arthritis. J. Cell. Mol. Med. 23 (9), 5876–5883. 10.1111/jcmm.14531 31313518 PMC6714168

[B34] Van der fits L. Mourits S. Voerman J. S. Kant M. Boon L. Laman J. D. (2009). Imiquimod-induced psoriasis-like skin inflammation in mice is mediated via the Il-23/Il-17 Axis. J. Immunol. 182 (9), 5836–5845. 10.4049/jimmunol.0802999 19380832

[B35] Wang W. Yu X. Wu C. Jin H. (2018). Differential effects of Wnt5a on the proliferation, differentiation and inflammatory response of keratinocytes. Mol. Med. Rep. 17 (3), 4043–4048. 10.3892/mmr.2017.8358 29286164

[B36] Yamanaka K. Yamamoto O. Honda T. (2021). Pathophysiology of psoriasis: a review. J. Dermatology 48 (6), 722–731. 10.1111/1346-8138.15913 33886133

[B37] Zhang C. Weng Y. Wang H. Zhan S. Li C. Zheng D. (2024). A Synergistic effect of triptolide and Curcumin on rheumatoid arthritis by improving cell proliferation and inducing cell apoptosis via inhibition of the Il-17/Nf-κb signaling pathway. Int. Immunopharmacol. 142 (Pt A), 112953. 10.1016/j.intimp.2024.112953 39226828

[B38] Zhang Y. Tu C. Zhang D. Zheng Y. Peng Z. Feng Y. (2015). Wnt/β-Catenin and Wnt5a/Ca pathways regulate proliferation and apoptosis of keratinocytes in psoriasis lesions. Cell Physiol. Biochem. 36 (5), 1890–1902. 10.1159/000430158 26202350

