# Supplementary figures and images for: Triptolide alleviates psoriasis through inhibiting the Wnt5a/β-Catenin signaling pathway
Source: Front Pharmacol. 2025 Apr 29;16:1534118. doi: 10.3389/fphar.2025.1534118 (PMC12069328; doi:10.3389/fphar.2025.1534118)

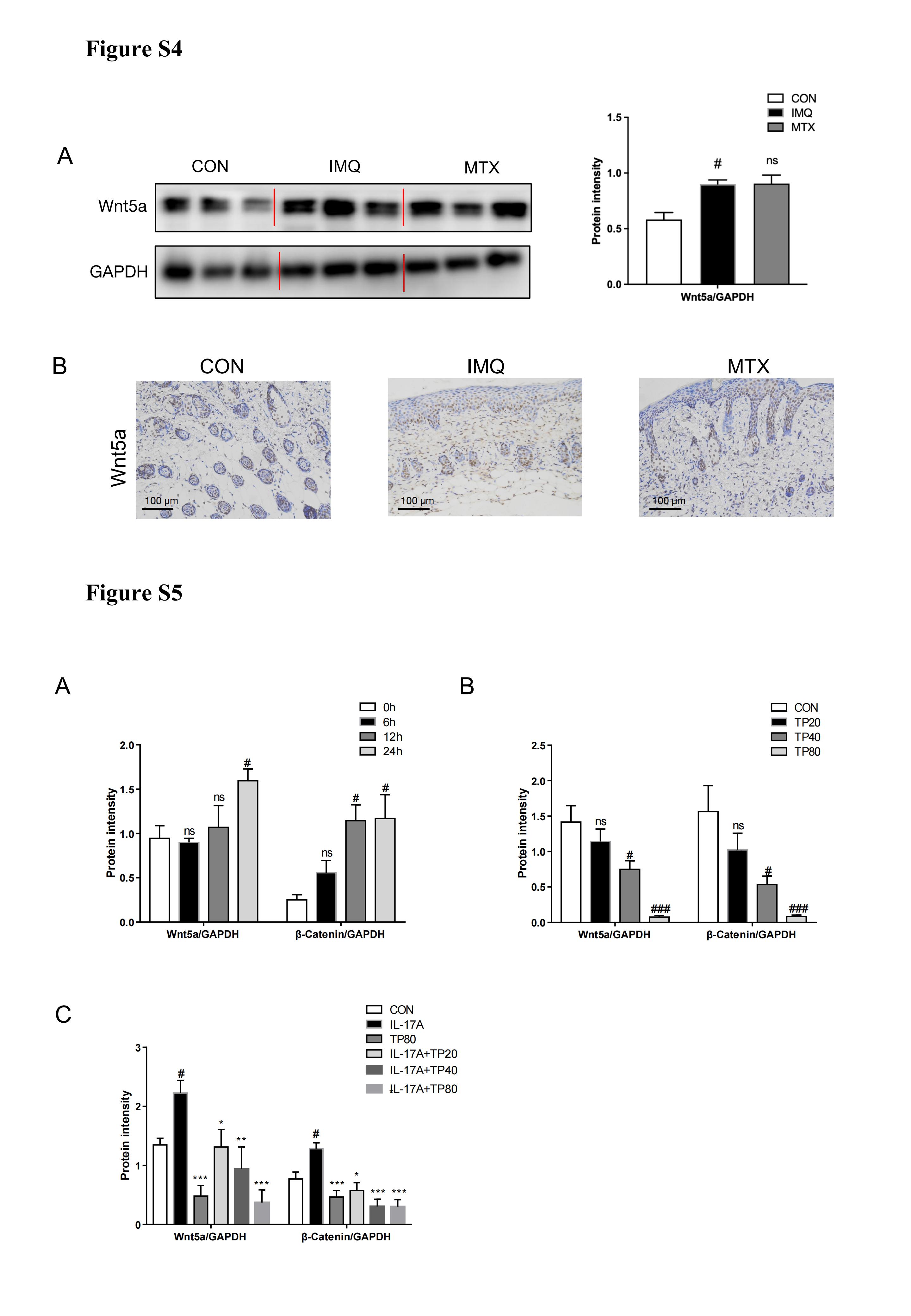

Supplement: Supplementary file 1 [file Image3.jpeg]

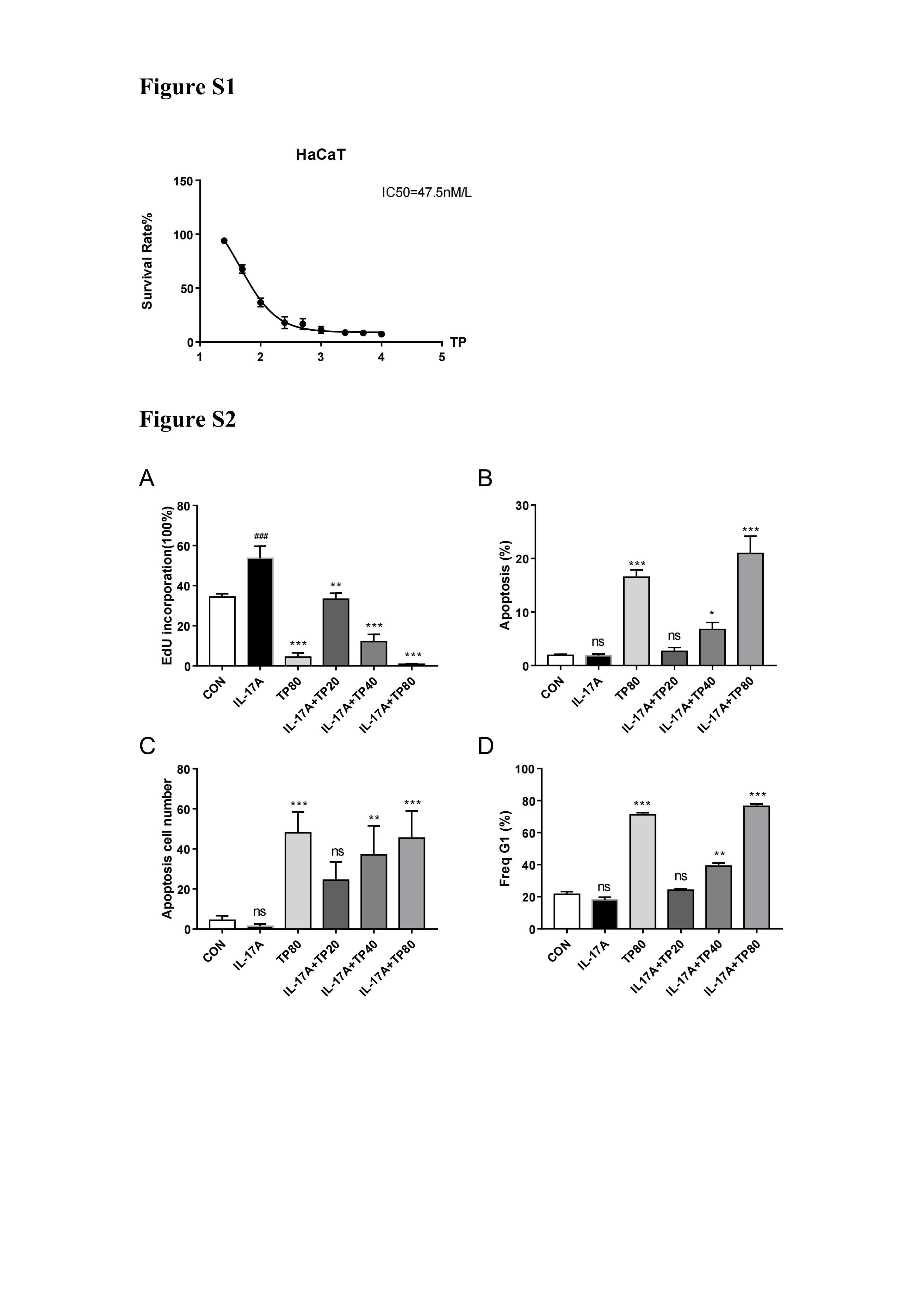

Supplement: Supplementary file 2 [file Image1.jpeg]

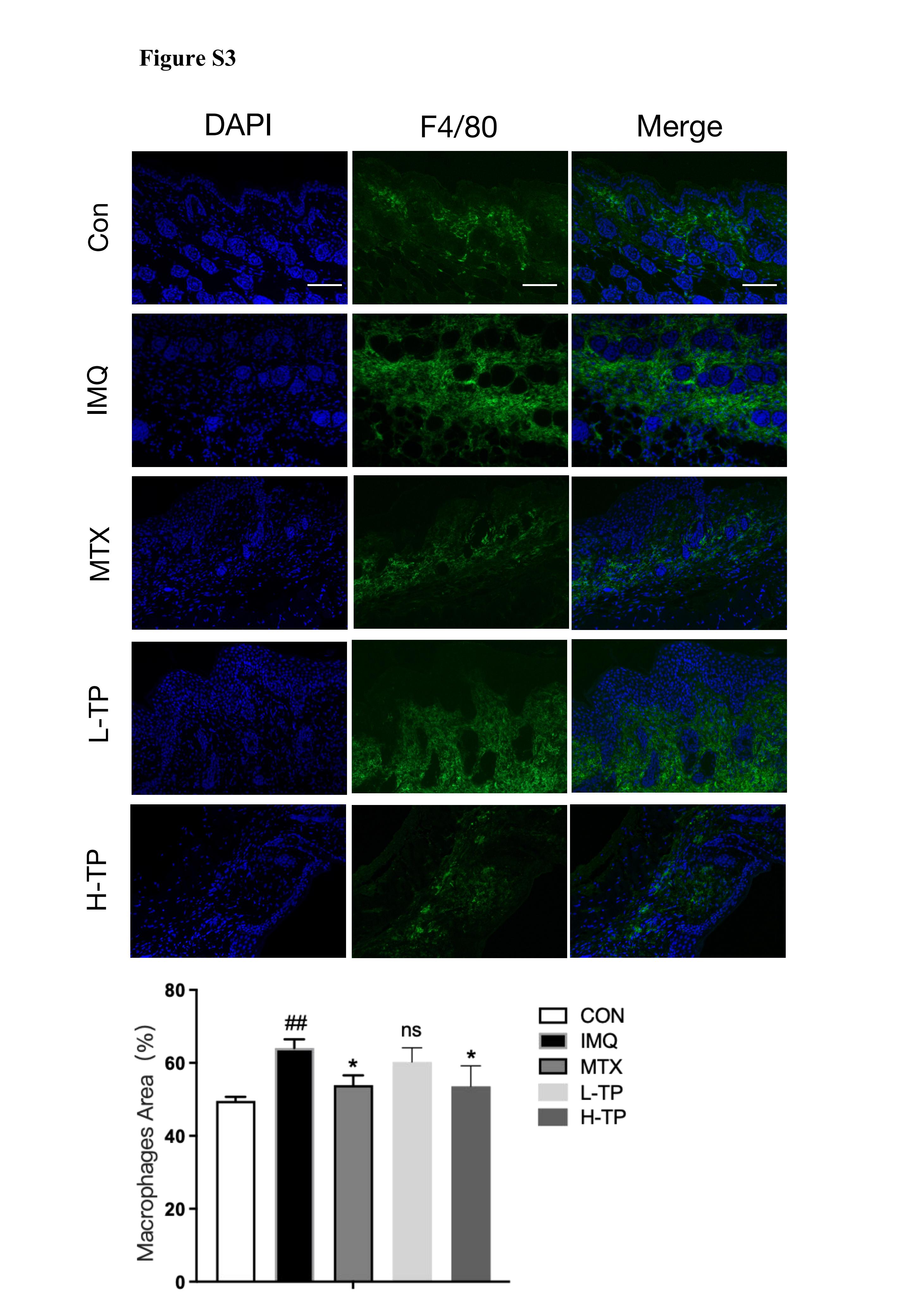

Supplement: Supplementary file 3 [file Image2.jpeg]
